# Comparison of Empirical and Reinforcement Learning (RL)-Based Control Based on Proximal Policy Optimization (PPO) for Walking Assistance: Does AI Always Win?

**DOI:** 10.3390/biomimetics9110665

**Published:** 2024-11-01

**Authors:** Nadine Drewing, Arjang Ahmadi, Xiaofeng Xiong, Maziar Ahmad Sharbafi

**Affiliations:** 1 Department of Human Science, Institute of Sport, Technical University of Darmstadt, 64289 Darmstadt, Germany; arjang.ahmadi@tu-darmstadt.de (A.A.); sharbafi@sport.tu-darmstadt.de (M.A.S.); 2 SDU Biorobotics, The Mærisk Mc-Kinney Møller Institute, University of Southern Denmark (SDU), 5230 Odense, Denmark; xizi@mmmi.sdu.dk

**Keywords:** wearable assistive device, exosuit, exo control, reinforcement learning, PPO

## Abstract

The use of wearable assistive devices is growing in both industrial and medical fields. Combining human expertise and artificial intelligence (AI), e.g., in human-in-the-loop-optimization, is gaining popularity for adapting assistance to individuals. Amidst prevailing assertions that AI could surpass human capabilities in customizing every facet of support for human needs, our study serves as an initial step towards such claims within the context of human walking assistance. We investigated the efficacy of the Biarticular Thigh Exosuit, a device designed to aid human locomotion by mimicking the action of the hamstrings and rectus femoris muscles using Serial Elastic Actuators. Two control strategies were tested: an empirical controller based on human gait knowledge and empirical data and a control optimized using Reinforcement Learning (RL) on a neuromuscular model. The performance results of these controllers were assessed by comparing muscle activation in two assisted and two unassisted walking modes. Results showed that both controllers reduced hamstring muscle activation and improved the preferred walking speed, with the empirical controller also decreasing gastrocnemius muscle activity. However, the RL-based controller increased muscle activity in the vastus and rectus femoris, indicating that RL-based enhancements may not always improve assistance without solid empirical support.

## 1. Introduction

Wearable assistive devices have become increasingly popular in recent years. They have been developed for several areas like the military [1], industry [2], and physical rehabilitation [3,4]. In the medical field, they are intended to help people enhance their activity and independence. Wearable assistive devices can initiate, support, or fully replace limb motor functions depending on the needs.

Among wearable assistive devices, exoskeletons are often used in rehabilitation [5,6] or supporting impaired people to regain their mobility [7]. These exoskeletons use rigid structures to support the body weight and apply the forces to support the movements of the joints. These structures are often heavy and can, when misaligned to the body, cause pain or injuries [8]. To address these problems, lighter assistive devices were developed. Commonly known as exosuits, these devices are made from soft fabric. There are several different approaches to designing soft exosuits, e.g., with pneumatic actuation [9], direct drive with the Bowden cable [10], or Serial Elastic Actuators (SEAs) [11,12].

To develop an effective controller for gait assistance that can support different users, one approach involves utilizing empirical and biomechanical data to create model-based approaches [13,14]. Empirical methods are often limited by human assumptions about optimal muscle behavior and gait patterns. While these controllers have proved to be safe to use in real applications like the ReWalk Exosuit [7], time-consuming tuning is often necessary to adjust it to different people and different tasks. Another approach is to use Reinforcement Learning (RL). Given the recent surge in artificial intelligence (AI) integration into daily life and human–machine interaction, AI is anticipated to significantly impact the field of human movement treatment and the control of assistive devices [15]. It has recently become more popular for developing and tuning controllers of assistive devices [16,17]. It is free from assumptions about muscle behavior and has the flexibility to learn directly from experience. Ref. [18] presents an RL-based controller developed with a deep neural network for a lower limb rehabilitation exoskeleton. Another example is the developed control for a hydraulic knee exoskeleton [19]. While those examples still rely on experimental data, ref. [20] developed a control purely based on simulation and Reinforcement Learning without experimental data. They achieved a significant reduction in metabolic costs for walking, running, and stair climbing. This aligns with the advancements in AI applications, particularly in enhancing the functionality and effectiveness of assistive technologies for individuals with mobility challenges. Ref. [21] shows an increase in the use of AI to create controls for exoskeletons, which create a significant reduction in metabolic cost like [20]. Often, the results are also better than other control strategies [18].

While recent studies have shown promising results using RL for lower-limb exoskeletons, many approaches rely heavily on simulation-based models and often struggle with real-world adaptation and generalization. Traditional empirical control methods, while effective, lack the flexibility needed to accommodate individual variability in users’ gait. These methods often require laborious manual tuning and fail to provide adaptive support tailored to the specific needs of each user. Given the limitations of current empirical and AI-based controllers, we aim to investigate which method provides superior assistance in terms of muscle activation and walking efficiency. Our approach directly compares an empirical controller—designed based on biomechanical data—with an RL-optimized control system.

In this study, we use a self-developed Biarticular Thigh Exosuit (BATEX) [11]. BATEX simulates the hamstring (HAM) and rectus femoris (RF) of the human leg with the help of SEAs and thus acts as artificial muscles that control the knee and hip joint. We apply both empirical and RL-based controllers on this exosuit. The controls developed in this study are based on the assumption that the change in the muscle length from the anterior muscles to the posterior muscles of the thighs can be used to generate muscle support during walking. Both controls have the same structure and are designed to be as simple as possible, which use force sensors in the artificial muscles, encoders for the motor position, and the Ground Reaction Force (GRF).

While a desired force curve for the empirical control is based on empirical data from the experiment, the parameters for the desired curve of the RL-based control are determined through Reinforcement Learning. During training, the simulation platform Scone [22] is used to find the best parameters to reduce the energy required for walking based on minimizing the muscle activation. Scone provides a framework for creating and optimizing models that are executed using underlying simulators like OpenSim. By using the same biarticular exosuit for both methods, we are able to make a fair comparison and filter out the strengths and limitations of each approach in providing adaptive, efficient walking assistance.

Given the complexity of human gait, particularly with biarticular actuators like those used in our exosuit, we expect the RL-based controller to outperform the empirical control. The ability of RL to learn nonlinear and multi-dimensional interactions between muscles and joints should lead to a more efficient, coordinated control strategy than the simpler empirical model. Additionally, previous research using PPO-based RL algorithms has shown significant reductions in metabolic cost for similar tasks [23]. We anticipate that the RL-based controller, by optimizing muscle activations over time, would be able to reduce the energy expenditure required for walking, making it superior to the empirical controller, which lacks such adaptive optimization. The reduction in energy expenditure can be related to muscle activation [24], even though there is no direct translation from activation to energy expenditure.

## 2. Materials and Methods

This section explains the walking experiments at two different speeds and the design of the empirical and RL-based controllers as well as the used exosuit.

### 2.1. BATEX Exosuit

**Figure 1:**
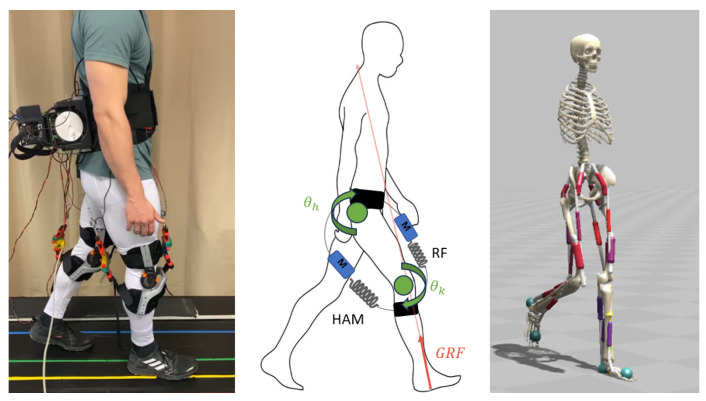
Exosuit and models of human and exosuit: the exosuit worn during an experiment (**left**). The schematic of the exo structure and functionality (**middle**). The neuromuscular model in Scone for the AI training (**right**).

BATEX is an assistive device developed to support human gait using a bio-inspired design. It was developed to investigate and analyze human gait. Using two SEAs, an attempt was made to mimic the functioning of the human rectus femoris (RF) and hamstring (HAM) muscles [13] actuating both the knee and hip joints. The structure is shown in Figure 1 with the motors on the back of the person on the left. These two muscles are important for balance as well as for the general movement of walking [25]. The SEAs can be adjusted and replaced by springs of different stiffness. It is possible to use the BATEX as a mono-articular actuator as well, while the biarticular version is used for this study. The SEAs are connected to steel cables, which are attached to the motor via a guide pulley. Both artificial muscles are then controlled by one motor—if the motor turns forward, the RF shortens and HAM becomes longer, and vice versa. Using encoders and force sensors, the exosuit can give feedback of the current state of the different parts. For further details, see [11].

### 2.2. Experiments

This study includes three phases of experiments: (1) identification of general control patterns, (2) walking at a fixed slow speed, and (3) walking at a preferred walking speed. We explain these three phases in the following.

*(1) Control identification:* The first experiment was carried out to record the change in the length of the artificial muscles. We conducted walking on the treadmill at a preferred speed while the subject wore the BATEX with low-stiffness springs (3 N/m) and blocked motors. This allowed the change in length in the two artificial muscles of BATEX to be determined using the force sensors (serial to spring). Although the exo force does not distort the user, the measured values can demonstrate the general pattern required for support. This way, the behavior of the artificial muscles can be observed and used for both controllers.

*(2) Slow walking at a fixed speed:* This experiment and the next one were performed with four healthy subjects (between 30±5 years old, 179±4 cm and 79±5 kg) to check the performance of both controllers. The muscle activities of the HAM, RF, vasti (VASs), gastrocnemius medialis (GAS), and soleus (SOL) were measured using surface electromyography (sEMG) sensors, and the GRF was recorded, as well as the force produced in the force sensors of the artificial muscles of the exosuit. In this experiment, the subjects were walking 7.5 min on a treadmill with a velocity fixed to 0.8 m/s.

*(3) Walking at preferred speed:* Every person has a preferred walking speed (PWS), in which the body potentially finds a balance between effort (energy consumption) and comfort. Primarily, we conducted experiments to find the subjects’ PWS. Using sEMG sensors, the same muscles as in the previous experiment were measured as well as GRF. For this, we first started walking at a moderate speed and increased the treadmill speed to detect the walking-to-running Preferred Transition Speed (PTS). Since the PWS is about 75% of the PTS [26], we used this estimate as an initial approximation of PWS. By slowly increasing and decreasing the treadmill’s speed according to the subject’s feedback, we found the PWS for each individual. Then, the main experiment of 7.5 min walking at this speed was conducted.

The second and third experiments were used to verify the functionality of the controls and to check if they support the wearer of the exosuit. According to [27], on average, 1.4 m/s is considered a comfortable walking speed. In [28], old people walk at an average speed of 0.6–1.2 m/s, while [29] mentions a range between 0.9 and 1.25 m/s. By choosing a speed of 0.8 m/s, we can cover general slow walking as well as the walking of elderly people. Walking slow can reduce the body’s stability [30], leading to less efficient energy expenditure. Ref. [31] found that the coordination is more complex and is more tiring for people. PWS in comparison is considered the most efficient way of walking [32] for each person. So, by choosing a very slow speed and PWS, we can compare the effect the controller has on inefficient and efficient walking. In both experiments, four trials were conducted: one case without an exosuit *NE* (No Exosuit), and three cases while wearing the exosuit, namely, *NC* (No Control), *Emp* (empirical control), and *RL* (RL-based control). To investigate the control effect on muscular effort, comparisons were made to the NC case, in which springs connecting the hip and knee were removed. So, the subjects walked with additional BATEX weight (4.5 kg) but without receiving support from actuators. The measurements taken without the exosuit were used as a reference. We expected to measure a reduction in the muscle activity of the measured muscles in the second experiment for both *Emp* and *RL* compared to *NC*. It was also expected that *RL* would reduce activity compared to *Emp*. In the third experiment, an increase in PWS was expected when using the two controls. If the exosuit supports the wearer, the wearer should have a higher PWS. Since we assumed that the RL-based control would provide better support, we expected *RL* to have the highest PWS.

### 2.3. Empirical Control

Based on the force measurements of the first experiment, a desired force curve was created for the empirical control that covers the whole gait cycle (from touch down until late swing). To be able to measure the created force in the springs connecting the hip and knee, the motors were blocked to make sure that the force would not be translated into a movement of the motor. The springs were chosen to have a low stiffness (3 N/m). It was chosen such that the subject was able to normally walk while the sensors in the spring were still able to measure the created force. The artificial HAM created forces between 6 N and 23 N during a whole gait cycle. The maximum force was measured at the beginning of the stance phase (0–16%) and at the end of the swing phase (83–100%) while the peak force was measured at about 92% of the gait cycle. At this moment, the leg was fully stretched and needed to be moved towards the body. The measured forces of the artificial RF ranged between 4 N and 22.5 N, while the highest force was measured during the late stance/start of the swing phase. Here, a maximum force of 22 N was measured. This is the phase right before and after the toe-off. While the leg moves backward, the HAM is slackening until it starts to swing forward. The highest force in the RF is measured right before the swing phase starts. The muscle activation measured with EMG during walking are similar for most healthy people [33]. BATEX uses two artificial muscles (RF and HAM) to create torque for supporting the wearer. Based on the similarities in the general walking behavior of different people, the torque pattern to support the wearer will also be similar between different subjects. Therefore, bases on the force measurements taken during the first experiment, we can define the desired exosuit forces. This allows a simple structure of the control, which can be directly compared to the RL-based control. As a desired force pattern to support the wearer actively, the artificial HAM should continuously be pulled during the stance phase, and the artificial RF should be pulled during the swing phase.

**Figure 2:**
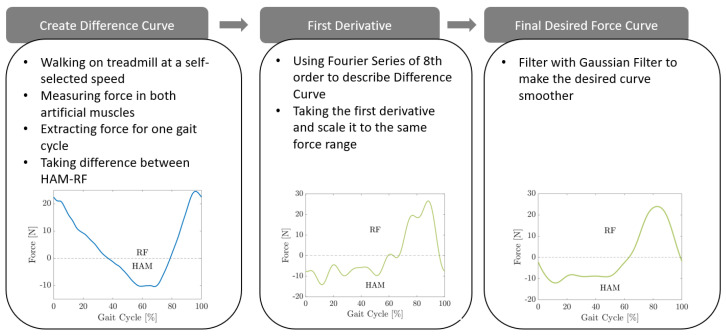
Development process of desired force curve.

To create a desired force curve with the desired pattern, the difference between the measured force of the artificial HAM and RF for one step was calculated, creating the U-shaped curve shown in Figure 2 in the first step of the workflow diagram. To find a formulation to generate the signal (instead of using a look-up table) that can be adaptable for different subjects and also for learning, we used a Fourier series of the eighth order to approximate this signal. The derivative of the HAM-RF difference can show us the previously mentioned desired force pattern with the right timing. Roughly speaking, a backward rotation of the motors resulting in HAM stretching in the stance phase and forward rotation generating RF stretching in the swing phase can be predicted by this signal. To create a smooth movement, the result was filtered using a Gaussian filter. To match the measured force range with the ones created by the desired curve, it was scaled down. The final result can be seen in Figure 2 in the last step. Since the stiffness of the springs of the exosuit can be exchanged, the desired force was not taken directly but, depending on which artificial muscle was active, transformed into the spring length using the respective stiffness. The control system uses the current measured forces, which are converted into spring lengths by subtracting the rest length of each spring. It then calculates the difference and compares it to the desired value. Finally, the relation of spring length to radius of the motor is used to calculate ΔΘ, which is the correction angle of the motor (see Equation (1)):

(1) $$\Delta \Theta =\left(\frac{F_{d}}{k}-\left(\frac{F_{HAM}}{k_{HAM}}-\frac{F_{RF}}{k_{RF}}\right)\right)\cdot \frac{1}{r_{m}}$$

**Figure 3:**
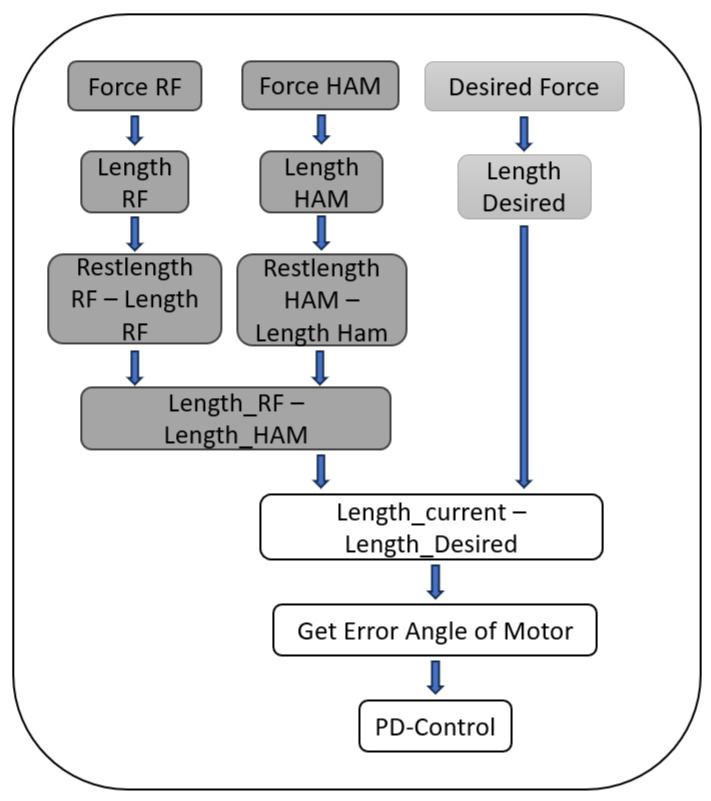
Control structure for both the empirical and the RL-based control.

Here, ΔΘ is the required motor rotation to generate, $F_{d}$ is the desired force and *k* represents the stiffness depending on the active artificial muscle. $F_{HAM}$ and $F_{RF}$ represent the current measured force in HAM and RF, respectively, and $k_{HAM}$ and $k_{RF}$ represent the respective stiffness of the SEAs. $r_{m}$ is the radius of the motor pulley radius. Afterwards, a Proportional–Derivative Control (PD-Control) was applied to the results before they were sent to the motor. We used a structure similar to that mentioned in [34] with an inner velocity loop. The parameters were set based on the hardware to avoid instability and allow for fast reaction for error correction. The whole structure of the control can be seen in Figure 3.

### 2.4. RL-Based Control

The RL-based control was developed based on the same control structure used for the empirical control (see Figure 2). A neuromuscular model provided by Scone was used and trained within an actor–critic learning environment. It was trained with Proximal Policy Optimization [35] to minimize the energy consumption of the muscles of the model during walking. The goal of the training was to (1) find the right muscle activation to make the model walk and (2) find the perfect parameters for the Fourier series of the eighth order—therefore to optimize the desired curve for the control. Both parts were trained simultaneously. We describe the model, the neural network, and the training algorithm in the following.

#### 2.4.1. Simulation Model

For training the RL, the model H0918M_osim4 provided by Scone was used. It has also been used in [36], which developed control strategies for different models (including the one we used in our study) based on RL. The model used is originally based on [37]. It is a simplified musculoskeletal model focusing exclusively on the lower body, omitting the arms and upper body musculature. It includes 18 muscles concentrated on the hip, knee, and ankle. This model is ideal for analyzing lower limb dynamics and optimizing gait without the complexity of upper body components. The included muscles were the iliopsoas, gluteus naximus, RF, VAS, HAM, biceps femoris, GAS, tibia anterior, and SOL. For simplicity, the two muscles vastus medialis and vastusus lateralis were combined into one. The same was performed with the hamstrings semitendinosus and semimembranosus. Because the biceps femoris is unlike the other two hamstrings, being a monoarticular muscle, it was included separately. The model used can be seen in Figure 1(right), which also represents the initial position for the learning. The starting position was chosen to be in the middle of a step. The desired curve was designed for one whole gait cycle. To ensure proper control, it is important to approximate the timing of the specific position within the cycle. This allows the control system to provide support at the correct moment during the gait. The duration was used to adapt the length of the curve to the necessary gait duration. During training, the adaption was performed by the average value of the last five detected steps. During training, a new step was detected when a foot touched again the floor and created a GRF of 30 N.

The exosuit was simulated by two coordinate actuators (CA) on each leg. They create forces at the hip and the knee to emulate the biarticular support provided by the BATEX. Since the motors simulated by the CAs would be attached outside the body, lever arms of 10 cm at the hips and 5 cm at the knees were selected to match the BATEX properties. In Figure 1(middle), the structure of the modeled exosuit is shown. The green circles represent the CAs, while the arrows show the direction of rotation. The artificial muscles of the exosuit are shown by the spring and the mass in blue connecting the hip with the knee. In every time step, the force of the HAM and RF of the model is measured. The difference between these two forces was compared to the desired value. The error between the current and the desired value was separated onto both actuators using the lever arms to achieve biarticular control. Afterward, a PD-control was used to control both CAs (similar to the empirical control).

#### 2.4.2. Network

The actor’s input vector has a length of 44 elements. It carries information about the fiber length of the muscles, the current muscle force, the orientation of the body, the velocity of the body, and the vertical GRF of the right and the left feet. Two hidden layers, each having a size of 64 neurons, and an output layer are separated into two parts: control parameters for the 18 muscles and the parameters for the developed control. The AI finds the right PD-Control parameters suitable for the simulation as well as the parameters for the desired curve.

The critic network also has four layers: the input layer, two hidden layers, and the output layer. The input and hidden layers are the same size as the actor network: 44 inputs and 64 hidden neurons per layer. The output layer has just one output.

#### 2.4.3. Training Algorithm

Training the AI is performed within 5 million runs. One run is finished either when the model ran ten steps or when the center of mass of the model is lower than 0.8, meaning instability. Each episode is a time step of 0.001 s.

In every episode, the RL agent collects the following information: the state at the beginning of the episode, the selected actions (activation of the model’s muscles and selected parameters for the Fourier series), the reward, the state after execution of the selected actions, the log-probabilities for both the muscle activation and the selected parameters combined, and the information of whether a solution is found or the run is aborted.

The learning of the policy is split into two parts: One part learns the parameters for the desired curve. The values change at the beginning of each run. The second part learns the activation of the 18 muscles. They are chosen in every time step. The actor and critic are updated every 2048 episodes. Randomization is used for the starting position and the angular velocity at the beginning of the training of a new run.

Updating the actor is performed using the Temporal Difference and the Generalized Advantage Estimation (TD+GAE) approach [38]. Mini-batches of the training data are used for efficient updates. They are shuffled to avoid sequential patterns. The policy is evaluated by computing the probability distribution of the chosen actions for the given states. Different methods are used to improve the learning: using entropy regularization prevents early convergence, while gradient clipping prevents excessively large updates that could disrupt learning. Additionally, the training data are reused ten times. This allows more stable learning and can improve convergence to a better policy [35]. The critic network is updated in the same rhythm as the actor network. Before using the loss to compute the gradients using back-propagation, the L2-Regularization [39] is used to prevent over-fitting. It penalizes the magnitude of the critic networks’ weights.

#### 2.4.4. Reward

The reward is chosen to minimize the energy expenditure for the model. It is based on the velocity of the model and the effort it takes to walk at that velocity. The effort is calculated based on the activation of the muscles (see Equation (2)). The higher the velocity and the lower the effort, the bigger the reward:

(2) $$\begin{array}{cc} reward= & v_{min}+\frac{\left(v-v_{min}\right)}{\frac{1}{18}\sum a}+r_{alive}\\ r_{alive}= & \left\{\begin{array}{c} 0,\mathrm{if}\;\mathrm{simulation}\;\mathrm{was}\;\mathrm{aborted}\\ 0.1,\mathrm{if}\;\mathrm{simulation}\;\mathrm{was}\;\mathrm{not}\;\mathrm{aborted} \end{array}\right. \end{array}$$

in which, *v* and $v_{min}$ are the current and minimum velocity (set to 0.2), respectively, and *a* denotes the muscle activation in the simulation model. Additionally, the reward is also increased by 0.1 for every time step the simulation is not aborted due to the falling of the model. This way, the model is motivated to actually move forward while reducing the needed activation for the muscles at the same time. Since the EMG sensors during the experiment measure the activation of the muscles, this way, the minimization in the model can be compared to the real-life reduction in muscle activation.

### 2.5. Simulation in Scone

Simulating the created torque produced by both developed controls (*Emp* and *RL*) within Scone using the same models as in the training of the AI shows the different effects they have on the muscles. The case *NE* is used for comparison, where the same model is used without CAs. The reflex model [40] is used for optimization adjusted to also activate the RF using PD-control and length feedback of the hamstrings based on [41]. Because the signal of the sEMG measures the neural activity of the muscle, which is comparable to the activation of the muscles, we compare the activation of the same muscles, which are also measured in the assisted walking experiment.

### 2.6. Adjustments for RL Outcome to Apply on Exosuit

In our RL-based approach, we do not consider any hardware limitations of the exosuit to gain a better understanding of the best possible adaptation of the exo control patterns to support humans. We scale the results down to our BATEX exo torque limitations to keep the identified patterns (instead of saturating them). This range complies with the one used for the empirical controller. We also apply another simulation round limiting the torque, and the overall solution is comparable to our scaled-down solutions. Here, we demonstrate the original unlimited RL-based outcomes that can be applied to other exoskeletons with higher torque generation capability in the future. In addition, physical constraints such as using one motor to actuate both HAM and RF artificial muscles are also considered to adapt the motor’s desired profile.

## 3. Results

In the following sections, the results of the simulation as well as the second and third experiments are presented. To be able to compare the different cases properly, first, the average step of each subject for each case is calculated and compared to the *NE* case. Afterward, the results are averaged using the results of all four subjects while eliminating the outliers. Now, having the different curves of each muscle for each case, the peak values and the Mean Average Value (MAV) are compared. As a baseline, the *NE* case is used. Both developed controls have a different impact on the measured activity of the different muscles. Statistical analysis is performed using ANOVA to assess group differences, followed by a post hoc test to identify significant pairwise differences between conditions. The resulting *p*-value from the ANOVA indicates the probability that the observed differences occurred by chance, with *p*-values below 0.05 considered statistically significant, suggesting a meaningful difference between the tested conditions. During slow walking, both controls reduce the muscle activity in the muscles at the back of the leg (HAM, GAS, and SOL) while causing higher muscle activity in the muscles in the front of the leg (RF and VAS). In the second experiment, the empirical control reduces the muscle activity in all muscles except SOL, while the RL-based control just reduces the activity in HAM and GAS and increases it in RF, VAS, and SOL. Both controls increase not just the PTS but also the PWS compared to the NC case. Details about the results of each experiment are presented in the following two paragraphs.

### 3.1. Simulation

**Figure 4:**
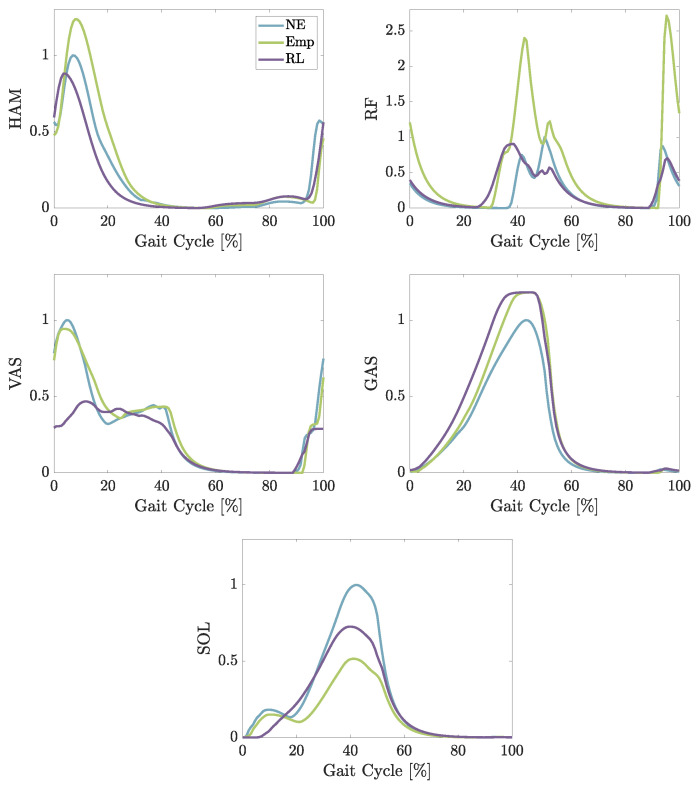
Average excitation of the muscles hamstring (HAM), vastus (VAS), gastrocnemius (GAS), and soleus (SOL) during the simulation of walking with different conditions: No Exo, Empirical, and RL-based control.

The simulation results show that both developed controls cause a change in the activation of the muscles (shown in Figure 4). The measured peak value of the activity in HAM is increased using *Emp*, while the duration of activation using *RL* is shortened. Also, the activation is doubled for RF using *Emp*. The *RL* control changes the behavior of RF. The muscles start earlier to activate during the late stance/early swing phase. While *Emp* does not change the muscle activation pattern of VAS noticeably, *RL* reduces the first peak by almost 50%. Afterward, it also follows the pattern created with *NE*. Both controls increase the activation for GAS by almost the same amount, while both reduce the activation for SOL. Here, *Emp* reduces the activation peak by almost 50%, while *RL* reduces it by about 25%. To confirm the observed positive effects of the *RL* control compared to *Emp* in these simulations, we design and conduct experiments 2 and 3 to verify the results.

### 3.2. Slow-Walking

**Figure 5:**
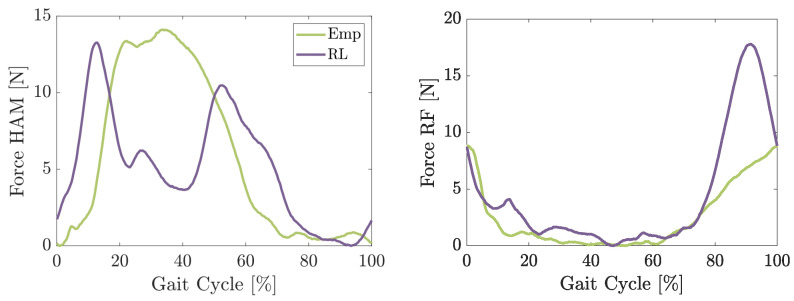
Measured Force with the force sensors of BATEX using the empirical- and RL-based control while walking at 0.8 m/s. The pre-tensioned force at rest position is subtracted.

During the first experiment, the average muscle force in the artificial muscles shows some difference (see Figure 5). The force in the HAM of the empirical control starts at about 10% to increase. It continues to pull until the early swing phase when it drops to almost zero. The force in the RF decreases in the early stance phase to almost zero and increases nearly linearly to around 8 N during the swing phase. The RL-based control creates a different curve. It starts earlier to create force in the HAM right at the beginning of the stance phase. The maximum force measured is similar to the force created with the empirical control. But it lasts just for about 20% of the gait cycle before it drops to about 5 N. At around 40% the second time, the force increases before it again falls to almost zero for the swing phase. But by the late swing phase, it already starts to increase again.

**Figure 6:**
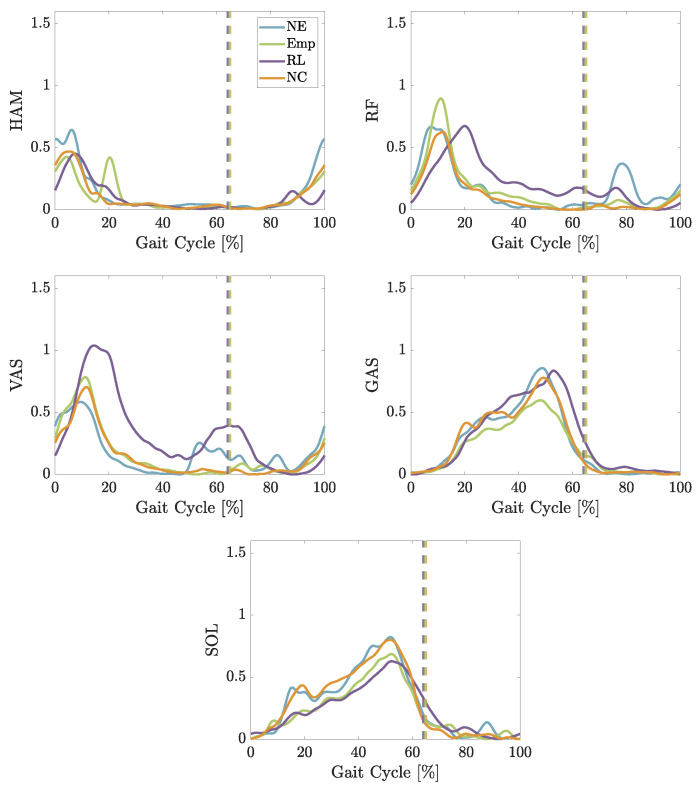
Average sEMG signal of four subjects measured during walking at 0.8 m/s with different conditions: No Exo, No Control, empirical and RL-based control. The vertical dashed lines show the take-off moment for different conditions.

**Figure 7:**
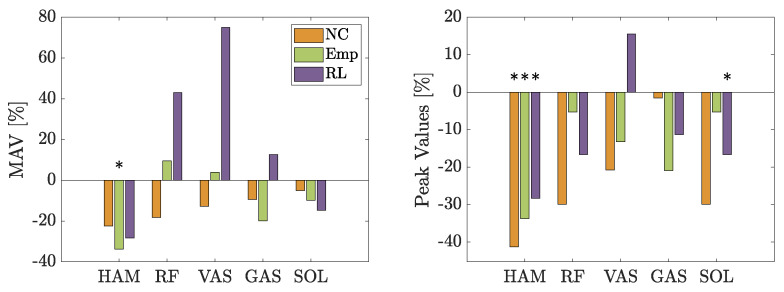
Change in the sEMG signal’s MAV and maximum values (peak) to measured sEMG signals without exosuit walking at 0.8 m/s. The asterix [*] denotes a *p*-value < 0.05.

Looking at Figure 6, both controls change the behavior of the muscles while walking at slow speed. Both the Mean Average Value (MAV) and the maximum values of the measured sEMG signal are compared to the case *NE* (Figure 7). In general, *Emp* decreases the measured activity in the muscles on the back of the leg. The reduction in MAV within HAM is significant with a *p*-value of 0.039. The MAV is reduced by the RL-based control in three muscles (HAM, GAS, and SOL) and increases the activity for VAS.

Regarding the maximum muscle activity values, both controls achieve lower values than *NE* in all muscles except for the RL-based control for VAS. The reduction in HAM is significant for the empirical control (*p*-value of 0.022), *NC* (*p*-value of 0.0363), and the RL-based control (*p*-value of 0.0125), while the reduction in SOL for the RL-based control is significant with a *p*-value of 0.039.

The duration of a step varies depending on the case: one step with *NE* in slow walking takes 1.333 s, and with *NC*, one step takes 1.340 s. Using *RL* extends the duration to 1.334 s, while using the *Emp* shortens the duration of a step to 1.309 s.

### 3.3. Walking at PWS

**Figure 8:**
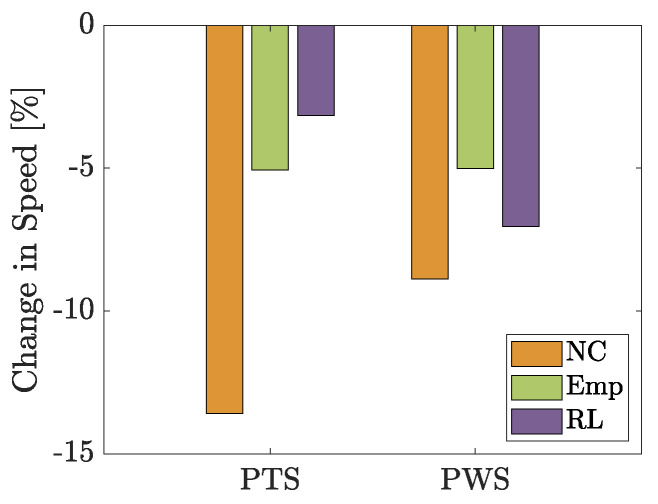
Changes in the average preferred walking to running transition speed (PTS) and preferred walking speed (PWS) compared to those of the No Exo case.

During the second experiment, the PTS and PWS of the subjects change depending on which control is used. Figure 8 shows, although no significance is found, both controls are able to increase the PTS and PWS compared to the case *NC*—in particular, the PTS is for *Emp* is about 2.5 times higher while for *RL* it is about 3 times higher. All the following results have to be seen in relation to the reduction in speed.

**Figure 9:**
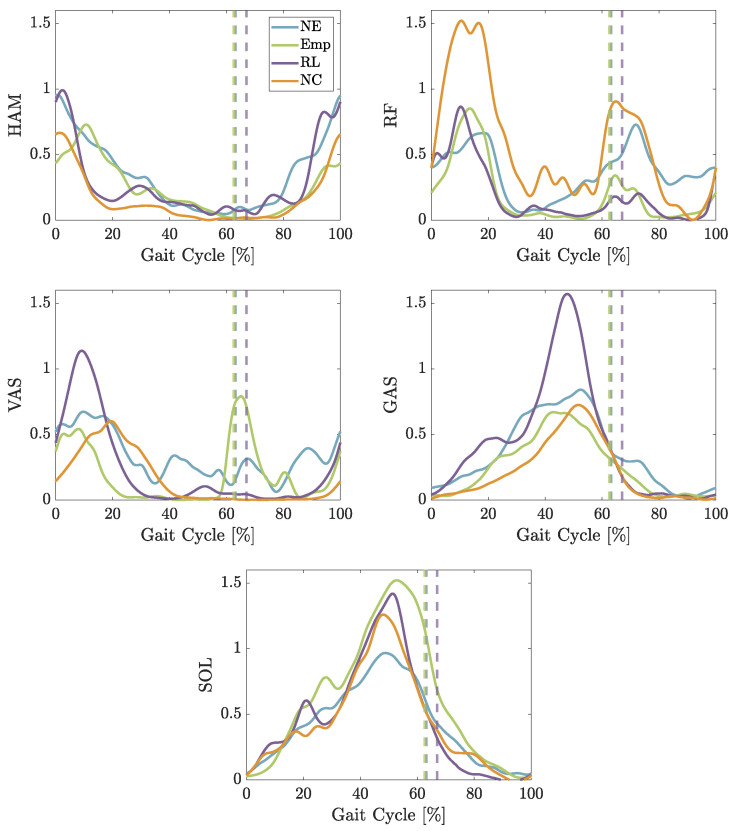
Average sEMG signal of four subjects measured during walking at preferred walking speed (PWS) with different conditions: No Exo, No Control, empirical and AI-based control. The vertical dashed lines show the take-off moment for different conditions.

As can be seen in Figure 9 the timing of the transition between the stance and swing phase changed compared to the first experiment. The empirical control causes a significantly later start of the swing phase compared to *NE* (*p*-value = 0.0037) and shortens the step duration on average by 8%. On average, the latest transition is measured using the RL-based control, which shortens the step duration by 6%.

The measured force during the second experiment is very similar to the one measured during slow walking. The main difference is that the force is increased for HAM to 15 N for the RL-based control and it drops down to about 10 N between 20% and 40% of the gait cycle. The force measured for RF does not change much besides the peak, being at around 15 N, decreasing a bit. The force measured using the empirical control within the artificial HAM is decreased to 10 N, and the force in RF in the early stance phase is increased to 11 N as well as in the late swing phase.

**Figure 10:**
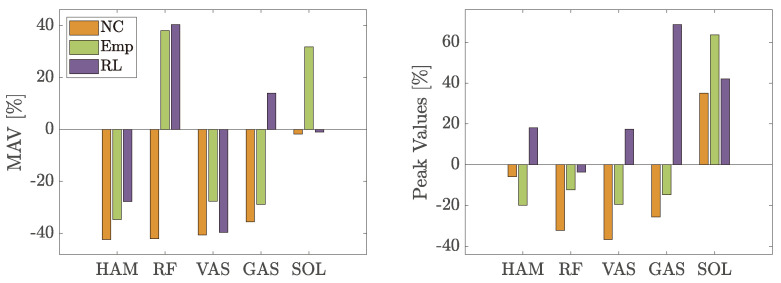
Change in % of the MAV of the sEMG signal and the peak value of the sEMG signal compared to measured sEMG signals without an exosuit while walking at a comfortable speed.

The results of the second experiment are slightly different from the first experiment. In Figure 10, the changes in MAV and the maximum value of the sEMG signals are shown. Using the empirical control reduces the MAV in all muscles but SOL. The largest reduction is measured in RF. The RL-based control reduces the MAV only in three muscles, namely, HAM, RF, and GAS. In all other muscles, it is increased.

Also, the timing of the transformation between the stance and swing phase changes. While all cases but RL-based control transform at around 61% of the step, using the RL-based control pushes the transformation to around 67%.

Unlike slow walking, the max values are not reduced in all muscles. While the empirical control is able to reduce the max value in all muscles except for SOL, the RL-based control just reduces the max value for HAM and RF. The empirical control reduces the max value in GAS significantly with a *p*-value of 0.007.

Also, while walking at PWS, the duration of the steps varies depending on the case. With *NE*, the subject’s steps take 1.054 s, and steps with *NC* take 1.089 s. Using *RL* extends the duration to 1.163 s, while *Emp* shortens the duration of a step to 1.048 s.

## 4. Discussion

The four key findings of this study, which will be elaborated on in this section, are as follows: (1) The experimental results are different from the simulation results. (2) The RL cannot improve the assistance quality of the empirical controller. (3) The support with both controllers is speed-dependent. (4) Both controllers can improve PWS and PTS speeds compared to the No Control case.

### 4.1. RL vs. Empirical Control

The hypothesis states that the two controls would reduce the subjects’ required effort during walking—while it is expected that the RL-based control would cause a greater reduction than the empirical control. Contrary to expectations, the RL-based control does not surpass the empirical control in reducing the required effort. This outcome aligns with the participants’ feedback, who deemed the empirical control more comfortable.

In slow walking, both controls are effective in modulating leg muscle activities: they reduce the MAV of leg muscles involved in knee flexion and foot lift, while increasing the activity of the leg muscles responsible for knee extension (see Figure 7). Moreover, both controls lead to a reduction in sEMG peak values across all muscles (except for VAS using RL-based control).

Specifically, the empirical control notably decreases the HAM muscle’s activation in slow walking during the early stance and late swing phases (see Figure 6) as evidenced by significant reductions in both peak and mean absolute values as shown in Figure 7. To understand the reasoning, we need to focus on the muscle behavioral changes in the swing phase. Even though the artificial RF is pulled in the late swing phase, the RF muscle engagement (visible from RF sEMG) does not change compared to not using the exosuit. The combination of physical RF muscle engagement and artificial RF pulling generates a faster forward movement of the swing leg. At the same time, HAM activity decreases during the late swing and the early stance. Thelen et al. have shown that stimulation of the hamstring muscles in the terminal swing or early stance phases can significantly increase the posterior pelvic tilt, indirectly influencing the acceleration and efficiency of gait [42]. Hence, the observed muscle engagement pattern alteration with the BATEX, particularly, the decreased HAM activity, could contribute to differing body accelerations during the stance phase. Consequently, the resulting movement has a different pace in the stance and swing phases compared to the *NE* case. The other effect of BATEX assistance relates to artificial HAM contribution in the mid- to late-stance phase. The provided support facilitates push-off, visible from reductions in the SOL and GAS muscles. Despite not significantly altering MAV or peak in the knee extensor and ankle plantar flexor muscle activity, it is perceptibly beneficial to participants due to their oral feedback. The HAM muscle’s capability to increase knee flexion and ankle dorsiflexion to support push-off shown by the artificial stimulation of this muscle during walking [42] aligns with our findings.

In slow walking, the RL-based control is able to achieve a comparable reduction in HAM sEMG to the empirical control during the stance phase and achieves an even greater reduction in the late swing (see Figure 7). In contrast to the peak value reduction, the MAV changes are not significant. At the same time, the amount of increase in other muscles (especially in RF, VAS, and GAS) is much higher in the RL-based control compared to the empirical control and even compared to the NC case. The increase in both RF and VAS starts around 15% of the gait cycle and lasts until the early swing phase. As can be seen in Figure 5, the RL-based controller starts pulling the HAM earlier than the empirical control and lasts longer until the early swing phase with a drop from 20% and 40% of the gait cycle. This suggests that the RL control’s unique timing and force application might require leg muscles to compensate for the forces created differently, especially the RF and VAS. During the swing phase, the exosuit creates higher forces using RL-based control in the artificial HAM compared to the empirical control. Creating a high force mid-swing and slowly reducing it until the late swing phase seems to support the movement more efficiently and leads to lower activity in both RF and VAS. Larger support of the swing leg provided by RL-control compared to the empirical controller yields lower activity in RF and VAS in late swing, which cannot be continued in the stance phase.

The effect of assistance at the preferred walking speed (PWS) is different from slow walking. The empirical control can reduce the MAV in all muscles except RF and SOL, where it is strongly increased instead, and all peak values except SOL (see Figure 10). Although not significant, the BATEX with empirical control supports the biological HAM in both the stance and swing phases. The stance and swing pace (acceleration/deceleration) adaptation paradigm with the BATEX found in slow walking does not exist with the PWS.

Walking at a higher speed reduces the negative influence of the RL-based control for RF and VAS (visible from RF sEMG), while MAV is comparable to slow walking in RF but is reduced in VAS. The reduction in SOL is smaller compared to slow walking. At the same time, the peak values in HAM, VAS, GAS, and SOL are increased. The only significant positive effect of RL-based control is in decreasing HAM and VAS MAV. Despite the increase in the RF muscle resembling that of slow walking, it is not significant in the PWS. The general trends shown in Figure 9 and Figure 10 show more benefit with empirical control than RL-based control for normal walking speed. Yet, due to substantial variations in individual speed preferences and movement adaptation, additional studies are essential to fully assess the potential of RL to enhance BATEX support at the PWS.

Despite the notion that increased muscle activation implies higher muscle force [43], this correlation is not straightforward outside of isometric conditions [24,44]. Alternatively, PWS could serve as an indicator of energy efficiency and control effectiveness. Both controllers (particularly the empirical one) demonstrate their capability to support the body effectively against the exosuit’s weight as evidenced by the PWS outcomes close to or even exceeding unassisted walking (*NE*) speeds for some subjects. While both controls improve PWS over uncontrolled conditions, the empirical control exhibits a more substantial impact, suggesting it may offer a more efficient support mechanism. However, comprehensive conclusions would benefit from further research involving a larger participant group.

### 4.2. Simulation vs. Experiments

The simulation can be compared to the first experiment, as the speed in the simulation is around 0.9 m/s. Comparing the muscle activation of the muscles, the simulation is able to create similar timing for all of the muscles except for RF (see Figure 4 and Figure 6). The measured RF in the experiments is active early-stance and late-stance/early-swing, while in the simulation, the main activation is shown in mid-stance until early-swing and late-swing. The simulation is optimized using a cost function to minimize the effort of the model. Since The co-contraction of muscles is not an efficient behavior, it is difficult to achieve [45,46]. Therefore, the co-contraction of RF with HAM in early-stance phase may lead to compensatory activation of RL and VAS in mid-stance. The pattern of GAS also shows higher activation in simulation during mid-stance while RF is active. This might be caused by the required higher force to flex the knee and plantar flex the ankle as the RF might contribute to knee extension at the same time. The GAS may compensate by increasing its activity to manage the dual demands of knee extension from RF and the knee flexion and ankle plantar flexion needed for propulsion.

The simulation predicts a reduction in activation for SOL for both controls, *RL* and *Emp*, as well as a later start of activation using the *RL* control. Comparing those results to the experimental data, the controls are both able to reduce the activation mid-stance. While the activation starts at the beginning of the early stance for *RL*, all other cases increase the activation stronger at the beginning of the step while *RL* slowly increases the activation. Although, the difference in reduction between the *RL* and *Emp* predicted in the simulation cannot be verified in the experiments, the predicted reduction in *RL* of around 60% can also be measured in the experiments.

The overall timing simulated for HAM matches the measured activation of the experiments. The *RL* is predicted to reduce the activation in the early stance and also to shorten the activation period, which can be verified during the experiments. The measured reduction in activation in late-swing during the experiments is not predicted by the simulation. This might be caused by the small reduction in RF activation during the late swing, which cannot be observed in the experiments. HAM is responsible for decelerating the swing movement to prepare for the touch down of the foot. As RF is responsible for hip flexion and knee extension, it can make sure the thigh will be lifting and supporting the preparation for the touch down of the foot. So, while the activation in the simulation is reduced, the HAM activation is not, while in experiments, the RF activation is not reduced but the HAM activation is.

Based on the simulation data, the RL algorithm successfully finds parameters to create a desired force curve that supports the walking of the model within Scone. However, when transferring a simulated control solution to an experimental setup, several challenges arise due to the idealized nature of simulations. Adjusting the scale of the applied forces and modifying the desired curve to match the hardware limitations does not seem to be sufficient to achieve the same level of performance observed in simulations. This discrepancy highlights the need for more sophisticated adjustments and fine-tuning to account for real-world variables and constraints that are not present in the simulated environment.

### 4.3. Limitations and Future Directions

The selected muscles for sEMG measurement are located near the points where the exosuit attaches to the leg, potentially leading to signal interference from the exosuit’s mechanical actions. To mitigate these movement artifacts on the sEMG sensors, we employ foam padding and straps to secure the sensors firmly against the skin. In our study, one participant exhibits substantially more sweating than others. Sweating can significantly influence sEMG signal quality and can reduce sEMG signal amplitudes. Our filtering algorithm aims to account for this variability but cannot eliminate all external factors. Expanding the study to include a larger participant pool could help mitigate these individual variations and provide a more robust understanding of sEMG signal behavior under various conditions.

The training model simplifies the structure of the exosuit using CAs actuated by a PD-control. In addition, a unique neuromuscular model with fixed parameters is used in the RL approach, and the result is applied to all subjects. A more precise model of the exo and an individualized model of the subjects can be implemented in a simulation model to improve the learning quality.

Both systems primarily aid the posterior leg muscles, but the AI control unexpectedly increases activation in the anterior muscles, especially VAS. This shift in muscle activation could lead to an altered muscular force balance with potential physiological implications if the exosuit is used consistently. Analysis of the average muscle activity suggests the empirical control performs better during the stance phase, whereas the AI control excels in the swing phase. Future research could explore a hybrid control approach, further AI training with adjusted parameters, prolonged training periods, or alternative exosuit simulations.

For conclusive insights, further testing with additional subjects is recommended to address possible variances, including subjects of different age groups. This way, it would be possible to also see the effect that the controls have on people who may have less muscle strength than the subject group we tested in this paper. A key limitation of our analysis is the confinement of the testing to controlled laboratory conditions, restricting the ability to evaluate the exosuit’s performance on diverse terrains and in real-world environments. Future studies would benefit from extended testing outside the lab, allowing assessment of the control strategies across various surfaces and under more dynamic, real-life conditions to better understand their robustness and adaptability.

Our RL-based simulations reveal how much of an increase in the torque limitations of the exo is required. Although applying high torque might also cause discomfort for the subject, this result could help us improve the mechanical design of the exo.

## 5. Conclusions

In this study, we compared the performance of an empirical control system and a Reinforcement Learning-based control for a bioinspired biarticular exosuit designed to assist human locomotion. Both control approaches were implemented using BATEX and tested on human subjects under different walking conditions.

The empirical controller, grounded in biomechanical data and human gait knowledge, demonstrated consistent reductions in muscle activation, particularly in the hamstrings and gastrocnemius muscles, showing effective assistance for leg muscles involved in walking. The RL-based controller, on the other hand, optimized parameters through training within a neuromuscular model and showed promise in learning complex interactions between muscles, especially in reducing hamstring activity. However, it unexpectedly increased activation in muscles like the vastus, indicating that RL-based methods may require further refinement to match the adaptability and efficiency of empirical methods in real-world scenarios.

Our findings highlight that while RL offers potential for personalized and adaptive control strategies, it may not consistently outperform empirical methods without a strong empirical foundation and careful tuning. The study suggests that combining the RL’s adaptive capabilities with empirical insights could enhance the performance of wearable assistive devices. Future work should explore hybrid approaches and longer training periods for RL, as well as testing across diverse populations to fully realize the benefits of AI-driven control systems in exosuits.

## Data Availability

The dataset used or analyzed during the current study is available under https://doi.org/10.48328/tudatalib-1571 (accessed on 24 October 2024).

## Abbreviations

The following abbreviations are used in this manuscript:

BATEX	Biarticular Thigh Exosuit
sEMG	Surface Electromyography
Emp	Empirical Control
GAS	Gastrocnemius
GRF	Ground Reaction Force
HAM	Hamstring
MAV	Mean Average Value
PPO	Proximal Policy Optimization
PTS	Preferred Transition Speed
PWS	Preferred Walking Speed
SEA	Serial Elastic Actutator
SOL	Soleus
VAS	Vastus
CA	Coordinate Actuator
NC	No Control
NE	No Exosuit
RF	Rectus Femoris
RL	Reinforcement Learning
